# Salivary Proteome, Inflammatory, and NETosis Biomarkers in Older Adult Practitioners and Nonpractitioners of Physical Exercise

**DOI:** 10.1155/2022/3725056

**Published:** 2022-04-23

**Authors:** Valeria B. Pacheco, Giulia Nery, Larissa L. Fernandes, Thais V. Santana, Manuel Jimenez, Leandro Borges, Elaine Hatanaka, Pedro Braga, Fernanda R. Monteiro, Jônatas B. Amaral, Luana S. Alves, André L. L. Bachi, Sérgio Gomes da Silva, Alexander Birbrair, Débora Heller

**Affiliations:** ^1^Post Graduate Program in Dentistry, Cruzeiro do Sul University, São Paulo 01506-000, Brazil; ^2^Core Facility for Scientific Research (CEFAP-Biomass), Universidade de São Paulo, São Paulo 05508-000, Brazil; ^3^Departamento de Didáctica de la Educación Física y Salud, Universidad Internacional de La Rioja, Logroño 26006, Spain; ^4^Interdisciplinary Program in Health Sciences, Institute of Physical Activity and Sports Sciences, Cruzeiro do Sul University, São Paulo 01506-000, Brazil; ^5^Federal University of São Paulo (UNIFESP), São Paulo 04021-001, Brazil; ^6^Department of Otorhinolaryngology, ENT Lab, Federal University of Sao Paulo (UNIFESP), São Paulo 04021-001, Brazil; ^7^Department of Restorative Dentistry, School of Dentistry, Federal University of Santa Maria (UFSM), Santa Maria 97105-900, Brazil; ^8^Post-Graduate Program in Health Science, Santo Amaro University (UNISA), Santo Amaro 04743-030, Brazil; ^9^Hospital do Câncer de Muriaé, Fundação Cristiano Varella, Muriaé 36880-000, Brazil; ^10^Department of Pathology, Federal University of Minas Gerais, Belo Horizonte 31270-901, Brazil; ^11^Experimental Research, Hospital Israelita Albert Einstein, São Paulo 05652-900, Brazil; ^12^Department of Periodontology, UT Health San Antonio, San Antonio 78229, USA

## Abstract

Since aging has been associated with increased production of inflammatory biomarkers, the ability to monitor older adults repeatedly is highly desirable, and saliva is an interesting biofluid for the search of biomarkers, as it is easily accessible in a noninvasive manner. However, given the incipient knowledge of salivary biomarkers in aging and its relationship to physical exercise, the present study is aimed at evaluating the protein expression and the levels of inflammatory and NETosis biomarkers in the saliva of practitioners (PE) and nonpractitioners (NPE) of physical exercise older adults. Six (6) practitioner and 4 nonpractitioner older adults were enrolled in this study. Unstimulated whole saliva was collected for analysis of the proteome by label-free mass spectrometry, as well as of the inflammatory status by evaluation of C-reactive protein (CRP), vascular endothelial growth factor (VEGF), and cytokines (TNF-*α*, interleukin- (IL-) 1*β*, and IL-8), while NETosis was assessed by myeloperoxidase (MPO) and neutrophil elastase. Regarding oral health, the decayed, missing, and filled teeth (DMF-T) index, bleeding on probing, suppuration, and probing depth measurement (mm) were evaluated. In addition, functional capacity was investigated using the General Physical Fitness Index (GPFI). In relation to the proteome analysis, 93 and 143 proteins were found exclusively in the PE and NPE groups, respectively; 224 proteins were common to both groups. Among these proteins, 10 proteins showed statistical difference (*p* < 0.05) between the groups: alpha-2-macroglobulin, component 3 of the complement, serotransferrin, and protein soluble in brain acid 1 were less expressed, while lactotransferrin, alpha-amylase 1, S100-A8, S100-A9, lactoperoxidase, and galectin-3 binding protein were more expressed in the PE group. No differences between groups were observed in the analysis of inflammatory and NETosis biomarkers. This study shows the potential utility of saliva for detecting protein biomarkers in a noninvasive biological sample of the elderly population.

## 1. Introduction

Although the aging process is not necessarily related to diseases and disabilities, chronic degenerative diseases, as well as subclinical inflammatory events named “inflammaging,” are often found during the aging process [1]. Particularly regarding the “inflammaging,” it is a phenomenon characterized by a chronic, sterile, and systemic low-grade inflammation associated with aging that can favour the development of several comorbidities and aging-related diseases [2, 3]. Beyond this information, *in vivo* research shows that neutrophil extracellular trap (NET) formation is also impaired with age, which could represent higher susceptibility to invasive bacterial disease in older adults [4, 5]. Thus, the current trend is to have an increasing number of older adults who, despite living longer, have greater chronic conditions and hospital incidents [6].

The increase in the number of chronic diseases is directly related to greater functional disability [7]. The impairment of the functional capacity of the elderly has important implications for the family, the community, the health system, and the life of the elders, as it causes greater vulnerability and dependence in old age, contributing to the decrease in well-being and quality of life of these individuals [7, 8]. There is evidence that regular exercise can minimize the physiological effects of a sedentary lifestyle and better regulate “inflammaging” [9]. Physical exercise, for instance, may increase the active life expectancy and limit the development and progression of chronic diseases and disabling conditions [10]. Consequently, the increase in life expectancy brings the need to add quality to the additional years of life, and the maintenance of good functional capacity is a fundamental part of this process. There is evidence that regular physical activity can be considered a valuable tool and low-cost alternative to minimize the deleterious effects of the aging process [11].

Saliva is a biofluid composed of exocrine secretion originating from the major (parotid, submandibular, and sublingual) and minor salivary glands, in addition to nonexocrine components such as epithelial and immune cells, microorganisms and their products, blood, and gingival crevicular fluid. Saliva contains biomolecules such as DNA, mRNA, microRNA, protein, and metabolites [12, 13] and has been recognized as a promising biological material for the early detection of diseases in general [14].

The circulating concentration of “inflammaging” markers has been evaluated in adults and older adults [15], and diverse molecules have been proposed as potential biomarkers [16]. However, there is no consensus on the best tools and/or biological samples to identify these markers. In this sense, saliva is an interesting biofluid for the search, as it is easily accessible in a noninvasive manner, it can be processed and stored easily, and it can be routinely obtained in clinical and home settings with minimal discomfort to the subject. However, to the best of our knowledge, aging biomarkers have not been analyzed in the saliva of older adults with different physical activity statuses.

Given the incipient knowledge of salivary biomarkers in aging and its relationship to physical exercise, the present study is aimed at evaluating the protein expression and the levels of inflammatory and NETosis biomarkers in the saliva of older adult practitioners (PE) and nonpractitioners (NPE) of physical exercise.

## 2. Materials and Methods

### 2.1. Ethics

The study was approved by the Universidade Cruzeiro do Sul ethics committee (3.045.972), and the experimental procedures were conducted according to the Declaration of Helsinki. All participants provided written informed consent before any assessment.

### 2.2. Participants

Older adult subject practitioners (PE, *n* = 6) and nonpractitioners of physical exercise (NPE, *n* = 4) aged 65 or over were recruited at the Science Center for Education and Physical Rehabilitation at Mogi das Cruzes, Brazil. Inclusion criteria comprised community-dwelling and autonomous older adults. As for the “practitioners” classification, the older adults had to perform 150 minutes of resistive exercise per week [17] (see Physical Exercise Program). Individuals with genetic, musculoskeletal, or uncontrolled endocrine diseases, who had chronic alcohol consumption or previous use of illicit drugs, and who used anti-inflammatory drugs, even for acute treatments for the past 6 months, were excluded from the study.

A single calibrated examiner (P.B.) was responsible to collect sociodemographic (sex, age, and education) and clinical data, as well as performing all physical activity assessments, described below.

### 2.3. Physical Activity Assessment

The volunteers were included in the PE group if they practiced regular physical exercise more than twice a week for, at least, 30 minutes. All physical activity parameters were assessed by the General Index of Functional Fitness (GPFI) [18], as proposed in the Senior Fit Test [19]. It is noteworthy to mention that GPFI is broadly used to evaluate the functional capacity through the tests: elbow flexion and sit-to-stand (to analyze muscular strength), timed up-and-go (TUG) (to assess sitting balance, transfer from sitting to standing), Gait Speed (GS) (to assess walking stability and changing gait course), and 6-minute walk (to verify aerobic capacity) [19]. The volunteers, who did not perform regular physical activity, were considered nonpractitioners of physical exercise.

### 2.4. Physical Exercise Program

The volunteers in the PE group were engaged in a program of exercise training that included both resistance and aerobic training. In relation to resistance training, all the participants performed it 3 times a week with submaximal intensity 14 on the Borg Subjective Scale (which represents “slightly tiring”) [20], and the training intensity was corrected every 15 days. In all sessions, they performed 3 sets of 10 repetitions of chest press, pulley, leg press, and gastrocnemius in extension, lumbar, and abdominal, and between each series, there was a 90-second rest period. On the other day, the participants were oriented to perform outdoor walking for, at least, 45-60 minutes.

### 2.5. Oral Health Assessment

One experienced calibrated dentist (V.B.P.) performed all oral health clinical measurements. To assess participants' accumulated history of dental caries, the decayed, missing, and filled teeth (DMF-T) index was used [21, 22]. Periodontal clinical parameters included bleeding on probing (BOP) and suppuration (SUP), in addition to the probing depth measurement (PD (mm)). These measurements were performed at 6 sites per tooth (mesiobuccal, buccal, distobuccal, mesiolingual, lingual, and distolingual) of all teeth, except for the third molars [23]. Periodontal clinical parameters were collected as they indicate potential intraoral sources of inflammation that, once expressed in saliva, could confound the assessment of the levels of inflammatory and NETosis biomarkers related to physical exercise.

### 2.6. Systemic Health Assessment

Anthropometric data (weight and height) was collected to determine the body mass index (BMI) by the following equation: BMI = weight/(height × height). Blood pressure and abdominal circumference were also collected for cardiovascular risk analysis [24].

### 2.7. Unstimulated Whole Saliva Collection, Processing, and Storage

Unstimulated whole saliva (UWS) samples were self-collected for protein, inflammatory, and NETosis biomarker analysis. Study participants were asked to refrain from eating and drinking, at least 1 h prior to saliva collection. To minimize circadian effects [25], UWS collection took place from 9:00 am to 11:00 am. Participants were instructed to remain seated and rest for a few minutes. Then, they let saliva accumulate on the floor of the mouth and let it passively flow from the lip directly into a sterile 50 ml collection tube for a period of 5 minutes. The flow rate (ml/min) was determined for each individual by recording the time and volume collected. The samples were kept on ice during the collection procedure and during transport to the laboratory. The tubes containing saliva were centrifuged using a Sorval RC 6+ centrifuge (Thermo Fisher, Waltham, MA) at 14000 × g for 20 min at 4°C. The whole saliva supernatant (WSS) resulting from this process was kept frozen at -80°C until analysis. The samples were stored for no longer than 3 months.

### 2.8. Proteome Analysis

Total protein concentration was assessed in duplicate using the bicinchoninic acid (BCA) assay (Pierce Chemical, Rockford, IL, United States). Twenty-five *μ*g of protein of each sample was separated for the enzymatic digestion of proteins. Briefly, HEPES buffer (pH 7.4, 0.1 M) was added to the samples; then, the protein disulfide bonds were reduced with DTT (dithiothreitol) (10 mM), and the samples were incubated for 60 min at room temperature. After reduction, the disulfide bridges were alkylated with iodoacetamide (40 mM) and incubated for 60 min at room temperature in the dark. The HEPES buffer was added again to adjust the pH, and finally, the proteins were digested enzymatically with trypsin at a ratio of 1 : 50 (*μ*g of enzyme to *μ*g of proteins) for 18 hours at 37°C. After obtaining tryptic peptides, the reaction was interrupted with 10% glacial acetic acid, and the peptide purification protocol was started for proteomic analysis.

The process of desalination and purification of the peptides was carried out using C18 resins coupled with tips. The solution containing the peptides was completely lyophilized on a rotary evaporator (Vacufuge™ Eppendorf AG, Barkhausenweg, Hamburg, Germany) for further analysis by mass spectrometry. The peptides obtained were resuspended in 0.1% formic acid and analyzed in an EASY-nano LC system (Thermo Scientific) coupled to an LTQ-Orbitrap Velos mass spectrometer (Thermo Scientific). The peptides were loaded onto a C18 column (PepMap, 75 *μ*m id × 10 cm, 3.5 *μ*m, 100 Å; New Objective, Ringoes, NJ, USA) and separated at a constant flow of 250 nl/min with an initial gradient of 100% mobile phase A (0.1% formic acid) to 34% mobile phase B (0.1% formic acid, 95% acetonitrile) in 60 min. After 60 min, the concentration of phase B goes from 34% to 95% in 15 min and remains at 95% of phase B for 5 min. The LTQ-Orbitrap Velos equipment was operated in a positive mode with a data-dependent acquisition (DDA) method.

The 20 most intense peptides were selected and fragmented by the CID (collision-induced dissociation) method. The raw files were accessed using the Xcalibur software and processed using the Proteome Discoverer (Thermo Scientific) 1.4.0.288, MaxQuant 1.6.1.0, and Perseus 1.5.8.5 software. Twenty raw files were obtained, which correspond to runs in technical duplicate of 5 replicates of the “sedentary” condition and 5 replicates of the “active” condition.

In Proteome Discoverer and MaxQuant software, the search was performed with the Homo sapiens database downloaded from Uniprot (http://www.uniprot.org). The 20 LFQ intensity files in the proteingroups.txt folder resulting from the MaxQuant search were loaded into Perseus. Firstly, a “reverse” and “potential contaminant” filter was used, and immediately after the initial filters, to have a better statistical result, the data were transformed with the application of log2 (*x*) In.

### 2.9. Cytokines and NETosis analysis

Salivary levels of MPO, elastase, VEGF, IL-8, TNF-*α*, CRP, and IL-1*β* (DuoSet Kit; Quantikine, R&D Systems, Minneapolis, MN, USA), as well as IL-6 and IL-10 (Invitrogen by Thermo Fisher Scientific, Vienna, Austria), were evaluated using the enzyme-linked immunosorbent assay (ELISA), following the manufacturer's instructions and as described previously [26]. A standard curve was built for each set of samples, and the biomarkers were assayed, yielding a correlation coefficient in the range of 0.98 to 0.99. The intra-assay and interassay coefficients of variance were 3–5% and 8–10%, respectively [27]. It is worth mentioning that the salivary concentrations of cytokines (IL-8, TNF-*α*, IL-1*β*, IL-6, and IL-10), CRP, MPO, elastase, and VEGF were normalized using the total protein concentration (Thermo Scientific Pierce™ BCA Protein Assay Kit, MA, USA).

### 2.10. Statistical Analysis

Data analyses were performed using Bioestat version 5.3 software (Bioestat, Instituto Mamirauá, Tefé, AM, Brazil), considering a significance level of 0.05. First of all, the data obtained here were compared with the Gauss curve with the normality determined through the Shapiro-Wilk test, followed by the homogeneity of variance analysis by the Levene test. Based on these procedures, the data presenting normality (parametric variables) was presented as the mean and standard deviation, and the data not presenting normality (nonparametric variables) was presented as a median and interquartile range. Therefore, to verify the occurrence of significant differences in sociodemographic, oral health, systemic, cytokines, NETosis, VEFG, and physical activity variables between the volunteer groups, it used not only Fischer's exact test for qualitative variables but also the Mann–Whitney test for quantitative variables. In order to guarantee a better statistical result in proteomic assessment, the data obtained were transformed by applying log2 (*x*). The samples categorized in “practitioners” (*n* = 6) and “nonpractitioners” (*n* = 4) groups were analyzed in duplicate and in two independent assays. After that, a filter of at least “5” valid values in each group was applied, which allowed us to reduce the number of valid values necessary to perform the statistical tests. Three statistical tests were performed: the first with permutation-based FDR (FDR = 0.05), the second with Benjamini-Hochberg FDR (FDR = 0.05), and the third with the *p* value, the *p* value threshold being equal to 0.05. The values were obtained from the MaxQuant version 1.6.1.0 results (MaxQuant, Max-Planck-Institute of Biochemistry, Munich, Germany) and processed in Perseus version 1.5.8.5 (Max-Planck-Institute of Biochemistry, Munich, Germany) with a valid value filter of at least 5 per group; that is, in the active group for 1 protein to be considered, it must appear in at least 5 samples of the 10 samples obtained, as they were in technical duplicate.

## 3. Results

### 3.1. Sociodemographic, Clinical, Physical Activity, and Oral Health Data

Sociodemographic data and results of oral, systemic, and physical evaluation of the participants are shown in Table 1. No statistical difference was observed in age, sex, education level, BMI, SBP, DBP, and cardiovascular risk (CR) between the groups. As expected, the GPFI showed a significant difference (median score in the PE group was 75.5 and in the NPE group was 43, *p* = 0.02).

Regarding oral health, participants pertaining to the NPE group were more severely affected by dental caries throughout life as they had a significantly higher mean DMF-T index and wore a dental prosthesis in at least one jaw more often than those pertaining to the PE group (100% versus 50%, respectively). Notwithstanding, when assessing the periodontal parameters (which were more comprehensively assessed in the PE group as they had more natural teeth), we observed that no participant had suppurating teeth, mean probing depths ranged from 1 mm to 1.31 mm, with a maximum percentage of bleeding sites of 8.33%, with no statistically significant difference between groups. The unstimulated salivary flow rate was considered normal for all adults (>0.1 ml/min) (Table 1).

### 3.2. Salivary Proteome

A total of 317 proteins were identified in the PE group, and 367 proteins were found in the NPE group. Among these proteins, 93 were exclusive to the PE group, whereas 143 were exclusive to the NPE, and 224 were common to both groups.

Table 2 shows the data concerning the 10 proteins that were statistically different (*p* < 0.05) between the volunteer groups. In addition, Figure S1 shows the unsupervised hierarchical clustering heat map related to this proteomic analysis, particularly presenting the data concerning the 10 proteins differentially expressed in the volunteer groups (NPE and PE).

It was observed that while alpha-2-macroglobulin, complement C3, serotransferrin, and protein soluble in brain acid 1 were less expressed, lactotransferrin, alpha-amylase 1, S100-A8, S100-A9, lactoperoxidase, and galectin-3 binding protein were more expressed in the PE group.

### 3.3. Inflammatory and NETosis Biomarkers

**Figure 1:**
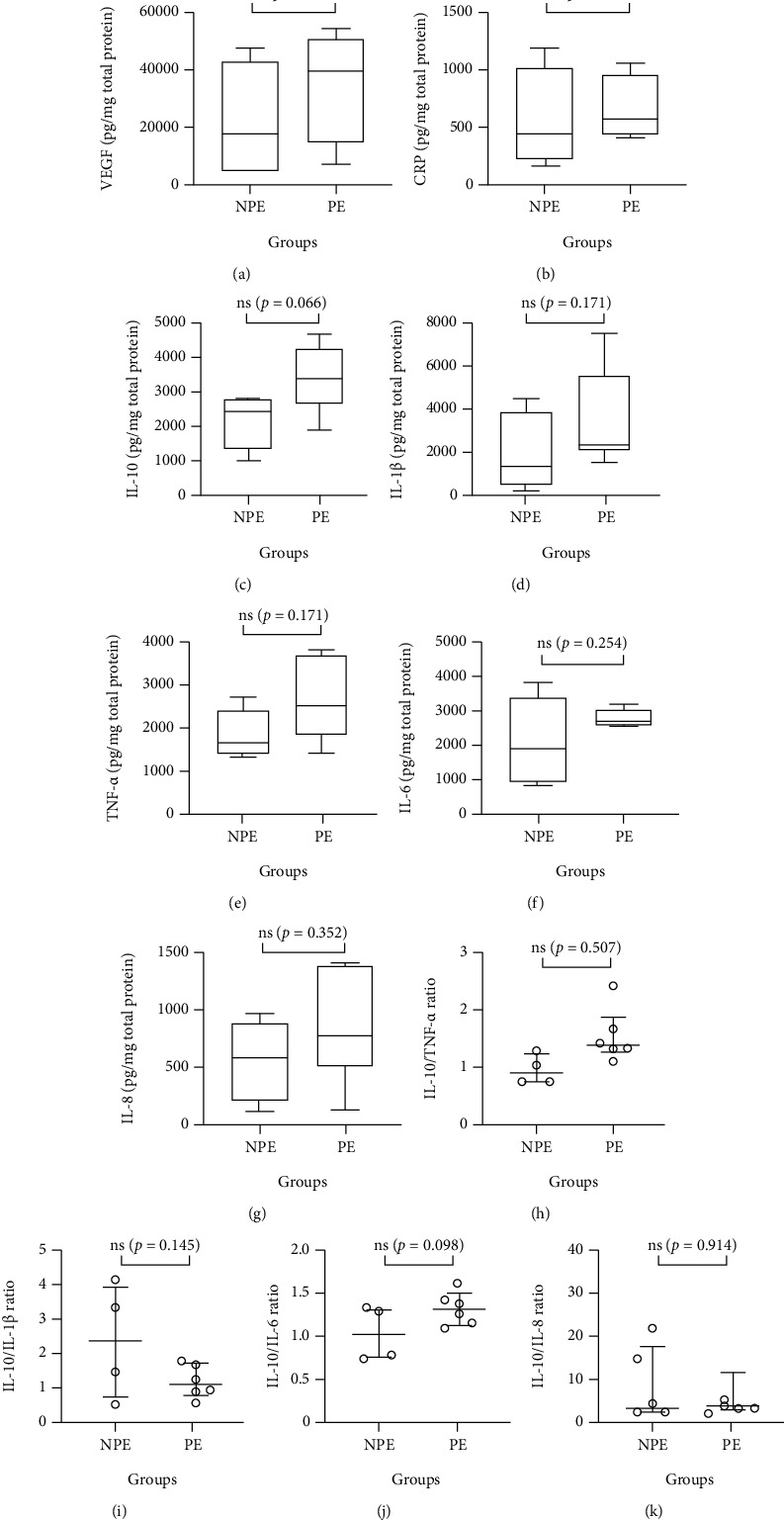
Levels of VEGF and inflammatory markers, as well as the ratio between anti-inflammatory (IL-10) and proinflammatory (TNF-*α*, IL-1*β*, IL-6, and IL8) cytokines in the saliva of nonpractitioners (NPE) and practitioners (PE) of physical exercise groups. VEGF (a), CRP (b), IL-10 (c), IL-1*β* (d), TNF-*α* (e), IL-6 (f), and IL-8 (g) were evaluated by the ELISA method. Data are shown as the median and interquartile range for 4–6 participants in terms of pg/mg of total protein.

Figure 1 shows the evaluation of the vascular endothelial growth factor (VEGF, Figure 1(a)) and also the inflammatory biomarkers (CRP (Figure 1(b)), IL-10 (Figure 1(c)), TNF-*α* (Figure 1(d)), IL-1*β* (Figure 1(e)), IL-6 (Figure 1(f)), and IL-8 (Figure 1(g)), as well as the ratio between the anti-inflammatory cytokine IL-10 and the other proinflammatory cytokines (IL-10/TNF-*α* (Figure 1(h)), IL-10/IL-1b (Figure 1(i)), IL-10/IL-6 (Figure 1(j)), and IL-10/IL-8 (Figure 1(k))) between the groups. Even though no significant statistical differences were found concerning these parameters, it is noteworthy to point out that salivary IL-10 levels showed a tendency to be raised in the PE group (*p* = 0.066, Figure 1(c)) as compared to the NPE group. Particularly, this elevation of IL-10 levels in saliva led to the increased IL-10/TNF-*α* ratio observed in the PE group in comparison to the values found in the NPE group (NPA, *p* = 0.0507, Figure 1(h)).

**Figure 2:**
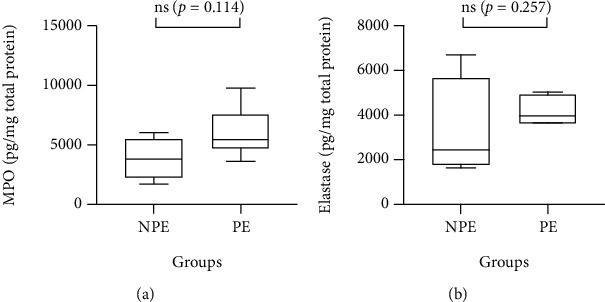
Levels of myeloperoxidase (MPO) (a) and neutrophil elastase (b) in the saliva of nonpractitioners (NPE) and practitioners (PE) of physical exercise groups. MPO (a) and neutrophil elastase (b) were evaluated by the ELISA method. Data are shown as the median and interquartile range for 4–6 participants in terms of pg/mg of total protein.

Figure 2 shows the results regarding MPO (Figure 2(a)) and neutrophil elastase (Figure 2(b)) analysis, and as described above, no significant differences between the volunteer groups were found (*p* > 0.05).

## 4. Discussion

Geriatric patients can often have several medical conditions that make blood collection difficult. Additionally, their veins become less elastic and can be easily injured or collapse during a venipuncture [28]. This study presents a novel, straightforward, and noninvasive approach using saliva for the assessment of biomarkers in this population. Since aging has been associated with increased production of inflammatory biomarkers, the ability to monitor older individuals repeatedly is highly desirable. Thus, saliva collections can be regularly performed and do not require complex or expensive procedures, so it is a promising source of protein biomarkers.

In this way, the present study applied an innovative approach aimed at identifying proteomic, inflammatory, and NETosis biomarkers in the saliva of older adult practitioners and nonpractitioners of physical exercise. Interestingly, the main result obtained was associated with the fact that 10 salivary proteins showed a significant difference between the groups, with 4 proteins being less expressed in the practitioners of the physical exercise group (alpha-2-macroglobulin, complement C3, serotransferrin, and protein soluble in brain acid 1), whereas 6 proteins (lactotransferrin, alpha-amylase 1, S100-A8, S100-A9, lactoperoxidase, and galectin-3 binding protein) were more expressed in the same group as compared to the data found in the nonpractitioners of the physical exercise group.

It is of utmost importance to highlight that the proteins less expressed in the practitioners of the physical exercise group have been reported as potential biomarkers for several pathologies. In fact, alpha-2-macroglobulin has been associated with an almost 3-fold greater risk of Alzheimer's disease [29]. Component 3 of the complement has been associated with age-related macular degenerative disease [30], ovarian cancer, and thyroid tumors [31]. Serotransferin has also been related to women with ovarian cancer [32]. Protein soluble in brain acid 1 has been linked to pancreatic cancer [33].

Among the proteins that were more expressed in the practitioners of the physical exercise group, lactotransferrin has bactericidal properties [34], S100-A8 protein has antimicrobial activity, and lactoperoxidase has antimicrobial properties, helping defend the host in the airways against infections [35], whereas galectin-3 binding protein is associated with the immune system, stimulating the host's defense against viruses and tumor cells [36, 37], in addition to participating in the regulation of inflammatory processes and the immune response [38]. Moreover, levels of lactotransferrin in saliva have been negatively associated with early diagnosis of Alzheimer's disease [39, 40], and a recent study showed that alpha-amylase was found in epithelial cells and appears to induce the proliferation and differentiation of these cells, particularly in the small intestine, maintaining a healthy intestinal environment [41].

It is known that reduction in physical activity or a sedentary lifestyle can lead to the appearance of health problems and anticipated physical frailty [42]. Here, we show that the individuals that practiced regular physical exercise presented higher levels of proteins with immunity and host protection functions.

Another aspect that also has been associated with the aging process is “inflammaging,” a phenomenon characterized by a gradual elevation of circulating concentration of inflammatory markers, such as CRP, TNF-*α*, and IL-6, in older adults (aged over 70 years), especially those who presented a sedentary lifestyle [6, 15, 43], even though other studies did not confirm these findings [44, 45].

Based on these pieces of information, the lack of statistically significant differences between volunteer groups in terms of inflammatory and NETosis salivary biomarkers not only corroborates our previous report [46] but also could be explained by the results obtained in the GPFI. In this respect, it is paramount to point out that although the practitioners of physical exercise presented a very good functional capacity, the nonpractitioners of the physical exercise group presented a regular functional capacity and not a sedentary profile or frail. Therefore, we can putatively suggest that a comparison of results obtained in a group with practitioners of physical exercise and another sedentary group could show significant differences. Corroborating this suggestion, our result concerning the evaluation of pro- and anti-inflammatory cytokine ratio, particularly IL-10/TNF-*α*, showed a strong tendency to be different between the volunteer groups (*p* = 0.0507, Figure 1(h)), with better control of inflammation in the PE group than the NPE group. According to the literature, evaluations of the ratio between anti-inflammatory and proinflammatory cytokines have been used as a crucial measure to characterize the inflammatory status in several body sites, including oral [47].

Oral health in the older adult's population can be determined by several factors such as age, gender, educational level, systemic conditions, or cognitive impairment [48]. There is substantial evidence of the bilateral link between oral and systemic diseases [49]; specifically, that periodontal disease is a risk factor for systemic conditions, such as cardiovascular disease [50]. Furthermore, impaired oral health has been associated with mastication and nutritional problems, especially among the elderly, with highly negative effects on their quality of life [51]. Periodontal diseases have also been associated with several other multifactorial inflammatory systemic disorders by mechanisms that may involve the dissemination of key periodontal pathogens, their virulence byproducts, and local inflammatory mediators to distant body sites through the circulatory system and/or the oral-gut axis [49, 52]. It becomes imperative the importance of evaluating the oral health of the older adult population in a comprehensive manner. The individuals included in the present study were independent and did not need the help of third parties for locomotion or self-care for oral health. Clinical data regarding dental caries was collected to describe the sample while periodontal parameters were collected to ensure that no bias was introduced in the study due to a possible uneven distribution of periodontal disease between groups. Although participants of the NPE group showed a greater caries experience (which is a conceivable finding considering their tendency to be older and less educated); both groups were similar in terms of periodontal parameters. This leads us to infer that any difference between groups regarding inflammatory and NETosis biomarkers, if detected, would not be attributed to intraoral sources. It is possible to state that the study participants had an oral health condition similar to the Brazilian elderly population [53, 54]. Furthermore, there was no difference in salivary flow rates between the groups. Although histological studies of the salivary glands show a reduction in the proportion of secretory cells, the aging process has very little effect on the salivary flow of healthy patients [48]. The presence of saliva is vital for maintaining oral health and adequate oral functions. The lack of saliva results in a rapid deterioration in oral health and has a negative impact on a patient's quality of life [55].

Coupled with the development of point-of-care technologies and the emerging trend of home screening for medical conditions, the clinical impact of scientifically credentialed salivary biomarkers for health/disease applications will improve the access to care, reducing health disparities and impacting healthcare worldwide [56]. Nonetheless, there are several challenges when using this biological fluid. The type of saliva collection, preanalytical procedures such as sample handling and processing, and other variables related to saliva collection and laboratory processing should all be considered very carefully while interpreting saliva biomarkers for the diagnosis of systemic diseases [13]. In spite of the progress in salivary proteomics, transcriptomics, and genomics, some of the biomarkers so far identified are not disease-specific, which will usually require more than one biomarker to fully enable saliva to be translated for personalized/precision medicine applications [57].

A limitation of our study was the small sample size, in addition to being a convenience sample and the inability to obtain proteomic data from one of the participants of the PE group. A larger sample is needed for a more comprehensive investigation. Additionally, further understanding of the role of the differently expressed proteins is needed to identify the major contributor(s) of aging and age-related diseases. Future studies should include longitudinal analyses as well as the evaluation of larger population samples, such as through the establishment of a saliva biobank.

## 5. Conclusions

The salivary proteome differed between older adult practitioners and nonpractitioners of physical exercise. The present study shows the potential utility of saliva as a noninvasive biological sample for detecting protein in the older adult population. Multidisciplinary approaches including salivary biomarkers are strongly desirable to pinpoint new strategies able to provide early diagnoses of age-related pathologies and provide a healthy lifespan for the aging population in the long run. This innovative study may result in the development of a better method for treating aging-related diseases and preventing or reducing the appearance of pathologies associated with the aging process.

## Figures and Tables

**Table 1:** Sociodemographic data and results of oral, systemic, and physical evaluation of the volunteer groups.

Parameters	Practitioners of physical exercise (*n* = 6)	Nonpractitioners of physical exercise (*n* = 4)	
	Median (IQR)	Median (IQR)	*p* value^∗^
*Sociodemographics*			
Age	67.5 (64.75-75)	78 (72.25-86)	0.11
Sex (*n*, %)			0.50
Male	5 (83.3%)	2 (50%)	
Female	1 (16.7%)	2 (50%)	
Education level (*n*, %)			0.19
<8 years of education	1 (16.7%)	3 (75%)	
≥8 years of education	5 (83.3%)	1 (25%)	
*Oral health*			
DMF-T	20.5 (14.5-22.5)	28 (28-28)	0.01
Bleeding on probing (%)	1.19% (0%-3.87%)	0% (0%-0%)^£^	0.57
Suppuration	0	0^£^	—
Probing depth (mm)	1.04 (1.03-1.34)	1 (1-1)^£^	0.29
Salivary flow rate	0.5 (0.25-0.82)	0.3 (0.3-0.75)	0.76
*Systemic health*			
Body mass index	27.9 (22.8-28.5)	25 (21.3-27.5)	0.61
SBP/DBP	135/79	120/75	0.48
Cardiovascular risk (*n*, %)			1.00
Ideal/acceptable	2 (33.3%)	2 (50%)	
Moderate/high	4 (66.7%)	2 (50%)	
*Physical activity*			
GPFI	75.75 (63.75-83.25)	43 (41.5-61)	0.02

IQR: interquartile range; DMF-T: decayed, missing, and filled teeth index; SBP: systolic blood pressure; DBP: diastolic blood pressure; GPFI: General Physical Fitness Index. ^£^Data pertaining to only one participant as the other three individuals in the NPE group had no natural teeth. ^∗^Fischer's exact test for qualitative variables (age, education level, and cardiovascular risk) and Mann–Whitney test for quantitative variables.

**Table 2:** Salivary proteins that differed between practitioners and nonpractitioners of physical exercise groups.

Accession number	Protein name	Student's *T*-test difference practitioners–nonpractitioners
P01023	Alpha-2-macroglobulin	-1.18361
P01024	Complement C3	-0.77063
P02787	Serotransferrin	-0.778669
P02788	Lactotransferrin	0.852229
P04745	Alpha-amylase 1	0.713232
P05109	Protein S100-A8	1.06714
P06702	Protein S100-A9	1.05926
P22079	Lactoperoxidase	0.56749
P80723	Brain acid soluble protein 1	-1.57619
Q08380	Galectin-3 binding protein	1.42149

Note: negative Student *T*-test values mean reduced expression, and positive values mean an increase in expression from practitioner elders.

## Data Availability

The data used to support the findings of this study are included within the article and the supplementary information file.

## Supplementary Materials

Supplementary Materials Table S1: salivary proteins identified in the subjects. Figure S1: representative graph (heat map) of the 10 proteins differentially expressed (*p* < 0.05) between practitioners (PE) and nonpractitioners (NPE) of physical exercise groups. Note: all the evaluations were performed in duplicate and are indicated by the letters a and b for each sample. In addition, it was not possible to obtain data from one of the participants in the PE group, and also, we added one more participant in the NPE group.
