# Supplementary material for: Salivary Proteome, Inflammatory, and NETosis Biomarkers in Older Adult Practitioners and Nonpractitioners of Physical Exercise
Source: Oxid Med Cell Longev. 2022 Apr 23;2022:3725056. doi: 10.1155/2022/3725056 (PMC9056209; doi:10.1155/2022/3725056)
Supplement: Supplementary Materials — Table S1: salivary proteins identified in the subjects. Figure S1: representative graph (heat map) of the 10 proteins differentially expressed (p < 0.05) between practitioners (PE) and nonpractitioners (NPE) of physical exercise groups. Note: all the evaluations were performed in duplicate and are indicated by the letters a and b for each sample. In addition, it was not possible to obtain data from one of the participants in the PE group, and also, we added one more participant in the NPE group. [file 3725056.f1.zip › 3725056.f1.pdf]

Table S1. Salivary proteins identified in the subjects.

Proteins present exclusively in practitioners of physical activity	Accession number	Protein name
	A0A024R9T1	HCG1745306, isoform CRA_a
	A0A087WUZ2	IgGFc-binding protein
	A0A087WWU8	Tropomyosin alpha-3 chain
	A0A0F7SZ86	IGHV2-70 protein (Fragment)
	A0A0I9QQP6	G protein-coupled receptor 154
	A0A0J9YY17	Dynein heavy chain 10, axonemal (Fragment)
	A0A0S2Z4I7	Vasodilator-stimulated phosphoprotein isoform 4 (Fragment)
	A0A1S5UZ39	Hemoglobin subunit alpha
	A0A1W6IYJ1	N90-VRC38.09 heavy chain variable region (Fragment)
	A0A286YF91	IgGFc-binding protein (Fragment)
	A0A2P9AHG4	Metallophosphoesterase
	A6XMH5	Beta-2-microglobulin
	A7E2D6	NAV2 protein]
	A8K0T9	cDNA FLJ75422, highly similar to Homo sapiens capping protein (actin filament) muscle Z-line, alpha 1, mRNA
	A8K739	cDNA FLJ77339
	A8MX94	Glutathione S-transferase P
	B0YJC5	Vimentin OS=Homo sapiens OX=9606 GN=VIM PE=1 SV=1 - [B0YJC5_HUMAN]
	B2R4F3	cDNA, FLJ92068, highly similar to Homo sapiens Rho GDP dissociation inhibitor (GDI) beta (ARHGDIB),mRNA
	B3KTM1	Malate dehydrogenase
	B4DHB3	Phosphoglycerate kinase
	B4DI38	Adenylyl cyclase-associated protein
	B4DI57	cDNA FLJ54111, highly similar to Serotransferrin
	B4DM82	Peptidyl-prolyl cis-trans isomerase
	B4DQ53	cDNA FLJ51275
	B4DU36	cDNA FLJ57168, moderately similar to Transcription factor HES-1
	B4DWQ3	Phosphoglycerate kinase
	B4E1B2	cDNA FLJ53691, highly similar to Serotransferrin
	B4E1B3	cDNA FLJ53950, highly similar to Angiotensinogen
	B4E1T6	cDNA FLJ54342, highly similar to Heat shock 70 kDa protein 1
	B4E397	cDNA FLJ55140, highly similar to SPARC-like protein 1
	B7Z747	cDNA FLJ51120, highly similar to Matrix metalloproteinase-9 (EC 3.4.24.35)
	B8ZZ51	Malate dehydrogenase, cytoplasmic
	C6GLU9	Uncharacterized protein

C9J1G2	DnaJ homolog subfamily B member 2 (Fragment)
D6RFL4	Monocyte differentiation antigen CD14 (Fragment)
D6RHX1	Mucin-7 (Fragment)
E5RFX6	Serine protease inhibitor Kazal-type 7
E5RJF6	Carbonic anhydrase 1 (Fragment)
E7ESK7	14-3-3 protein zeta/delta (Fragment)
E9PN95	Uteroglobin
F6KPG5	Albumin (Fragment)
F8VV32	Lysozyme
F8WE70	Serpin B13
G3V1R1	Basic salivary proline-rich protein 1
G3V361	Calmodulin-1 (Fragment)
G8I0M6	Alkaline phosphatase liver/bone/kidney (Fragment)
H3BQN4	Fructose-bisphosphate aldolase
H3BRL0	Bardet-Biedl syndrome 2 protein homolog
H3BU78	Fructose-bisphosphate aldolase (Fragment)
H7C0X8	Macrophage-capping protein (Fragment)
H7C234	Dual-specificity phosphatase 28 (Fragment)
H7C3U4	E3 ubiquitin-protein ligase MYCBP2 (Fragment)
H7C5U6	Ribonuclease 3 (Fragment)
I3L1H9	Zymogen granule protein 16 homolog B (Fragment)
I3L312	Protein disulfide-isomerase (Fragment)
I3L3P5	Protein disulfide-isomerase (Fragment)
J3QKP1	Cadherin-1 (Fragment)
J3QSF7	Myeloperoxidase (Fragment)
K7EMV3	Histone H3
P00338	L-lactate dehydrogenase A chain
P00491	Purine nucleoside phosphorylase
P01857	Immunoglobulin heavy constant gamma 1
P01859	Immunoglobulin heavy constant gamma 2
P04083	Annexin A1
P06899	Histone H2B type 1-J
P0DOY2	Immunoglobulin lambda constant 2
P14618	Pyruvate kinase PKM
P31151	Protein S100-A7
P35908	Keratin, type II cytoskeletal 2 epidermal
P52209	6-phosphogluconate dehydrogenase, decarboxylating
Q13747	Alpha-1 antitrypsin (Fragment)
Q14019	Coactosin-like protein
Q16378	Proline-rich protein 4
Q2EQ98	Envelope glycoprotein gp160

	Q2Q9B7	Glucose-6-phosphate 1-dehydrogenase (Fragment)
	Q499Z3	Schlafen-like protein 1
	Q49AK0	LTA4H protein
	Q5JPJ9	Uncharacterized protein DKFZp686D0114
	Q5SX91	Rab GDP dissociation inhibitor (Fragment)
	Q658Z1	Uncharacterized protein DKFZp666B131 (Fragment)
	Q6PEJ8	HP protein
	Q8IUI4	Putative protein SNX29P2
	Q8IZK6	Mucolipin-2
	Q8TCG4	TPMsk1 (Fragment)
	Q8WUW7	Pyruvate kinase (Fragment)
	Q96BG6	ACTN4 protein (Fragment)
	Q9GZL9	Beta-globin (Fragment)
	Q9HCC1	Single chain Fv (Fragment)
	Q9NSB8	Homer protein homolog 2
	Q9UM85	Beta-globin protein (Fragment)
	V9HW35	Epididymis secretory protein Li 55
	W8QEY1	Lactoferrin OS=Homo sapiens
**Proteins present exclusively in nonpractitioners of physical activity**	**Accession number**	**Protein name**
	A0A024R755	Calumenin, isoform CRA_a
	A0A068LKQ8	Ig heavy chain variable region (Fragment) OS=Homo sapiens OX=9606 PE=2 SV=1 - [A0A068LKQ8_HUMAN]
	A0A075B6R9	Immunoglobulin kappa variable 2D-24 (non-functional) (Fragment)
	A0A087WTV4	Cleavage and polyadenylation-specificity factor subunit 1 (Fragment)
	A0A0A0MRZ8	Immunoglobulin kappa variable 3D-11
	A0A0A0MS15	Immunoglobulin heavy variable 3-49
	A0A0C4DGB6	Serum albumin
	A0A0G2JL54	Complement C4-B
	A0A0G2JRN3	Alpha-1-antitrypsin
	A0A0U4DKQ8	Haptoglobin (Fragment)
	A0A0X1KG69	InaD-like protein
	A0A0X9V9B3	MS-F1 light chain variable region (Fragment)
	A0A125U0V1	MS-F1 heavy chain variable region (Fragment)
	A0A140T9Y7	tRNA (cytosine(34)-C(5))-methyltransferase
	A0A193CHR0	10E8 heavy chain variable region (Fragment)
	A0A1W2PQK0	Protein SCO2 homolog, mitochondrial (Fragment)
	A0A2P9AHE7	Copper-exporting P-type ATPase A
	A0A2P9AJL7	Acetolactate synthase large subunit IlvX

A0A2P9ANQ8	Putative protease, membrane anchored]
A0A2P9APB3	Putative soluble lytic murein transglycosylase
A0M8Q6	Immunoglobulin lambda constant 7
A2J1N7	Rheumatoid factor RF-ET10 (Fragment)
A2J1N9	Rheumatoid factor RF-ET12 (Fragment)
A8K2U0	Alpha-2-macroglobulin-like protein 1
A8KAK1	cDNA FLJ77398, highly similar to Homo sapiens UDP-glucose ceramide glucosyltransferase-like 1, transcript variant 2, mRNA
A8MZG1	Thrombospondin-1 (Fragment)
B0AZS6	14-3-3 protein zeta/delta
B2D098	Alpha-1 antitrypsin Valcamonica variant (Fragment)
B2R6X5	cDNA, FLJ93166, highly similar to Homo sapiens heat shock 70kDa protein 6 (HSP70B') (HSPA6), mRNA
B2R8I2	cDNA, FLJ93914, highly similar to Homo sapiens histidine-rich glycoprotein (HRG), mRNA
B3KTT7	Pannexin
B3KUV6	cDNA FLJ40727 fis, clone TKIDN2000127, highly similar to Glycogenin-2 (EC 2.4.1.186)
B4DEF7	cDNA FLJ60062, highly similar to 78 kDa glucose-regulated protein
B4DI39	cDNA FLJ54328, highly similar to Heat shock 70 kDa protein 1
B4DI70	cDNA FLJ53509, highly similar to Galectin-3-binding protein
B4DL17	cDNA FLJ52558, highly similar to Keratin, type I cytoskeletal 13
B4DNK4	Pyruvate kinase
B4DRR7	cDNA FLJ59399, highly similar to Keratin, type II cytoskeletal 4
B4DRY0	cDNA FLJ54379, highly similar to Keratin, type II cytoskeletal 6E
B4DWU6	cDNA FLJ51361, highly similar to Keratin, type II cytoskeletal 6A
B4E022	cDNA FLJ56274, highly similar to Transketolase (EC 2.2.1.1)
B4E1S8	cDNA FLJ59147, highly similar to Cysteine-rich secretory protein 3
B4E1V0	cDNA FLJ54839, highly similar to Lactotransferrin (EC 3.4.21.-)
B5BUM9	Kallikrein 13 (Fragment)
B7Z1L2	cDNA FLJ59586, highly similar to Diacylglycerol kinase zeta (EC 2.7.1.107)
B7ZAS5	cDNA, FLJ79289, highly similar to Extracellular matrix protein 1
B8ZWD8	Diazepam binding inhibitor, splice form 1D(1)
B8ZZL6	Macrophage-capping protein (Fragment)
C8CHS3	Truncated extracellular matrix protein 1
C9J386	Histone H2A

D3DP13	Fibrinogen beta chain, isoform CRA_e
D3DQX7	Serum amyloid A protein
E1B2D1	Hemoglobin alpha-1 globin chain variant (Fragment)
E5RFU9	Serine protease inhibitor Kazal-type 5
E7EQB2	Lactotransferrin (Fragment)
E9M4D4	Hemoglobin alpha-1 globin chain (Fragment)
E9PNW4	CD59 glycoprotein
F8VNQ3	Teneurin-2
F8W696	Apolipoprotein A-I
G3GAU4	Anti-H1N1 influenza HA kappa chain variable region (Fragment)
G3V3E6	ERO1-like protein alpha
G5D862	Cytochrome c oxidase subunit 1
H0YAL1	Centrosomal protein of 63 kDa (Fragment)
H0YEG8	Nucleobindin-2 (Fragment)
H0YLF3	Beta-2-microglobulin (Fragment)
H3BMQ8	Fructose-bisphosphate aldolase A (Fragment)
H3BRB8	Protein SOGA3 (Fragment)
H3BVC8	Prostasin (Fragment)
H7C5H1	Complement factor B (Fragment)
I3L223	Leucine-rich repeat-containing protein 59
I3L4E5	Aldehyde dehydrogenase, dimeric NADP-preferring (Fragment)
I3NI44	TOM1-like protein 1
J3KRE2	Rho GDP-dissociation inhibitor 1
J3KTJ1	Myosin regulatory light chain 12A (Fragment)
J3QR68	Haptoglobin (Fragment)
K7EJB9	Calreticulin (Fragment)
K7EJY8	Galectin-3-binding protein (Fragment)
K7EN27	Protein/nucleic acid deglycase DJ-1 (Fragment)
K7ESM3	Galectin-3-binding protein (Fragment)
L8E7V1	Alternative protein SPTLC1
O14597	Folate binding protein (Fragment)
O14942	Heat shock protein beta (Fragment)
O60218	Aldo-keto reductase family 1 member B10
P03973	Antileukoproteinase
P04259	Keratin, type II cytoskeletal 6B
P04792	Heat shock protein beta-1
P07237	Protein disulfide-isomerase
P08670	Vimentin
P0DOX6	Immunoglobulin mu heavy chain
P14550	Alcohol dehydrogenase [NADP(+)]

P14780	Matrix metalloproteinase-9
P29762	Cellular retinoic acid-binding protein 1
P36952	Serpin B5
P50395	Rab GDP dissociation inhibitor beta
P80188	Neutrophil gelatinase-associated lipocalin
P80723	Brain acid soluble protein 1
Q0QET7	Glyceraldehyde-3-phosphate dehydrogenase (Fragment)
Q13727	AHNAK-related protein (Fragment)
Q14CN2	Calcium-activated chloride channel regulator 4
Q2TUW9	Lactoferrin
Q4QQH3	SLC8A1 protein (Fragment)
Q53HF2	Heat shock 70kDa protein 8 isoform 2 variant (Fragment)
Q59FC2	Acyl-Coenzyme A dehydrogenase family, member 8 variant (Fragment)
Q5T123	SH3 domain-binding glutamic acid-rich-like protein 3
Q5ZEZ4	Alpha-actinin 1 (Fragment)
Q68CZ6	HAUS augmin-like complex subunit 3
Q68D45	Uncharacterized protein DKFZp686C09257
Q6MZV6	Uncharacterized protein DKFZp686L19235
Q6N041	Uncharacterized protein DKFZp686O16217 (Fragment)
Q6NS95	IGL@ protein
Q6UWP8	Suprabasin
Q6ZVX7	F-box only protein 50
Q7Z3Z0	Keratin, type I cytoskeletal 25
Q86TT1	Full-length cDNA clone CS0DD006YL02 of Neuroblastoma of
Q86TY5	Galectin
Q8IZU1	Protein FAM9A
Q8WYJ6	Septin-1
Q96BQ1	Protein FAM3D
Q96K68	cDNA FLJ14473 fis, clone MAMMA1001080, highly similar to Homo sapiens SNC73 protein (SNC73) mRNA
Q9NP55	BPI fold-containing family A member 1
Q9NPP6	Immunoglobulin heavy chain variant (Fragment)
Q9UI81	PRO0159
Q9UIV8	Serpin B13
Q9UK54	Hemoglobin beta subunit variant (Fragment)
Q9UL71	Myosin-reactive immunoglobulin heavy chain variable region (Fragment)
Q9UL72	Myosin-reactive immunoglobulin heavy chain variable region (Fragment)
Q9Y6R7	IgGFc-binding protein

	R4GMQ5	Cathepsin B (Fragment)
	S6B291	IgG H chain
	V9HW05	Epididymis luminal protein 210
	V9HW34	Epididymis luminal protein 213
	V9HW68	Epididymis luminal protein 214
	X6R3S7	Trefoil factor 3
**Proteins present in both groups**	**Accession number**	**Protein name**
	A0A024R528	Interleukin-1
	A0A024R609	Pyruvate kinase
	A0A024RC30	Desmoglein 3 (Pemphigus vulgaris antigen), isoform CRA_a
	A0A075B6H7	Immunoglobulin kappa variable 3-7 (non-functional) (Fragment)
	A0A087WWF8	Mucin-like protein 1
	A0A087WYF5	Salivary acidic proline-rich phosphoprotein 1/2 (Fragment)
	A0A0A0MRQ5	Peroxiredoxin-1
	A0A0B4J1R6	Transketolase
	A0A0C4DGM6	Mucin-21
	A0A0C4DGN4	Zymogen granule protein 16 homolog B
	A0A0G2JMB2	Immunoglobulin heavy constant alpha 2 (Fragment
	A0A0J9YXP8	Glucose-6-phosphate isomerase (Fragment)
	A0A0K0K1H8	Epididymis secretory sperm binding protein Li 71p
	A0A0U1RR93	SH3 and multiple ankyrin repeat domains protein 3
	A0A0X9T0H6	GCT-A5 heavy chain variable region (Fragment)
	A0A0X9T7V9	GCT-A4 light chain variable region (Fragment)
	A0A0X9TD47	MS-D1 light chain variable region (Fragment)
	A0A0X9UWL5	GCT-A5 light chain variable region (Fragment)
	A0A125U0U7	MS-C1 heavy chain variable region (Fragment)
	A0A193CHQ9	10E8 heavy chain variable region (Fragment)
	A0A1L2BPJ2	Kallikrein-1 isoform 4
	A0A1U9X7W4	HSPA1A
	A0A1W6IYI5	N90-VRC38.08 heavy chain variable region (Fragment)
	A0A1W6IYJ0	N90-VRC38.03 heavy chain variable region (Fragment)
	A0A2P9AST2	Uncharacterized protein
	A0M8Q9	C1 segment protein (Fragment)
	A0NA61	Codes for truncated alpha mRNA of alpha heavy chain disease patient LTE (Fragment)
	A2J1N0	Rheumatoid factor RF-IP14 (Fragment)
	A2KBB9	Anti-(ED-B) scFV (Fragment)
	A2MYD4	V2-7 protein (Fragment)

A2MYE1	A30 (Fragment)
A8K008	Uncharacterized protein
A8K486	Peptidyl-prolyl cis-trans isomerase
A8K4L6	Vang-like protein
A8K5I6	cDNA FLJ78643, highly similar to Homo sapiens cornulin (CRNN), mRNA
B1AN48	Small proline-rich protein 3 (Fragment)
B2R4M6	Protein S100
B2R7Z6	cDNA, FLJ93674
B3EWG6	Protein FAM25G
B3KPZ8	cDNA FLJ32530 fis, clone SMINT2000185, highly similar to TRANSKETOLASE (EC 2.2.1.1)
C9JEV0	Zinc-alpha-2-glycoprotein
C9JKF7	Lymphocyte-specific protein 1 (Fragment)
C9JL93	Serpin B13 (Fragment)
D3DP16	Fibrinogen gamma chain, isoform CRA_a
D6CHE9	Proteinase 3
D6RD17	Immunoglobulin J chain (Fragment)
D6RE86	Ceruloplasmin (Fragment)
D6REL8	Fibrinogen beta chain
E7ER44	Lactotransferrin
E9PK47	Alpha-1,4 glucan phosphorylase
E9PKG6	Nucleobindin-2
E9PLJ3	Cofilin-1 (Fragment)
F2Z393	Transaldolase
F5H386	Lactoperoxidase
F5H6Q0	Rho GDP-dissociation inhibitor 2 (Fragment)
F8WBR5	Calmodulin-2
F8WE65	Peptidyl-prolyl cis-trans isomerase
G3V1A4	Cofilin 1 (Non-muscle), isoform CRA_a
B3KS49	cDNA FLJ35478 fis, clone SMINT2007796, highly similar to Gelsolin
B3KVV6	cDNA FLJ41607 fis, clone CTONG3001370, highly similar to Homo sapiens alpha-2-macroglobulin-like 1 (A2ML1), mRNA
B4DE36	Glucose-6-phosphate isomerase
B4DEK5	cDNA FLJ54596, highly similar to Proactivator polypeptide
B4DFN9	cDNA FLJ54303, highly similar to Heat shock 70 kDa protein 1
B4DJI1	cDNA FLJ52549, highly similar to L-lactate dehydrogenase A chain (EC 1.1.1.27)
B4DMB4	Synaptobrevin-like 1, isoform CRA_a
B4DMJ7	HCG2015269, isoform CRA_c

B4DN40	cDNA FLJ54368, highly similar to Phosphoglucomutase-2 (EC 5.4.2.2
B4DNL5	Protein disulfide-isomerase
B4DNY3	Adenylyl cyclase-associated protein
B4DQ50	cDNA FLJ56823, highly similar to Protein-glutamine gamma-glutamyltransferase E (EC 2.3.2.13)
B4DRT4	cDNA FLJ51535, highly similar to Phosphatidylethanolamine-binding protein 1
B4DS71	cDNA FLJ57081, moderately similar to WD repeat protein 1
B4DTI0	cDNA FLJ52521, highly similar to Actin-like protein 3
B4DVQ0	cDNA FLJ58286, highly similar to Actin, cytoplasmic 2
B4DVZ8	Leukotriene A(4) hydrolase
B4DW52	cDNA FLJ55253, highly similar to Actin, cytoplasmic 1
B4DWS9	cDNA FLJ57640, highly similar to Serpin B5
B4DWU0	cDNA FLJ56791, highly similar to Keratin, type I cytoskeletal 16
B4E1M1	cDNA FLJ60391, highly similar to Lactoperoxidase (EC 1.11.1.7)
B4E216	cDNA FLJ57339, highly similar to Complement C3
B4E2U0	6-phosphogluconate dehydrogenase, decarboxylating
B4E3A8	cDNA FLJ53963, highly similar to Leukocyte elastase inhibitor
B7Z4U6	cDNA FLJ55803, highly similar to Gelsolin
B7Z507	cDNA FLJ51036, highly similar to Matrix metalloproteinase-9 (EC3.4.24.35)
B7Z553	cDNA FLJ51266, highly similar to Vitronectin
B7Z565	cDNA FLJ54739, highly similar to Alpha-actinin-1
B7Z5V2	cDNA FLJ54141, highly similar to Ezrin
B7Z6P1	cDNA FLJ53662, highly similar to Actin, alpha skeletal muscle
B7ZAL5	cDNA, FLJ79229, highly similar to Lactotransferrin (EC 3.4.21.-)
B7ZLF8	Uncharacterized protein
B7ZW15	Uncharacterized protein
C7DJS2	Glutathione S-transferase pi (Fragment)
C8C504	Beta-globin
G3V5B3	ERO1-like protein alpha (Fragment)
H0YAS8	Clusterin (Fragment)
H0YFX9	Histone H2A (Fragment)
H0YGG5	Alpha-2-macroglobulin-like protein 1 (Fragment)
H0YJV2	Serine palmitoyltransferase 2 (Fragment)
H3BPS8	Fructose-bisphosphate aldolase A (Fragment)

H3BUH7	Fructose-bisphosphate aldolase A (Fragment)
H6VRF8	Keratin 1
H6VRG2	Keratin 1
I3L3W9	Aldehyde dehydrogenase, dimeric NADP-preferring (Fragment)
I3NI03	Protein disulfide-isomerase (Fragment)
J3QLC9	Haptoglobin (Fragment)
K7EM49	6-phosphogluconate dehydrogenase, decarboxylating (Fragment)
K7EQQ3	Keratin, type I cytoskeletal 9
L0R4T3	Histone H2B
O75594	Peptidoglycan recognition protein 1
P00558	Phosphoglycerate kinase 1
P00738	Haptoglobin
P01023	Alpha-2-macroglobulin
P01024	Complement C3
P01034	Cystatin-C
P01036	Cystatin-S
P01037	Cystatin-SN
P01591	Immunoglobulin J chain
P01833	Polymeric immunoglobulin receptor
P01860	Immunoglobulin heavy constant gamma 3
P01861	Immunoglobulin heavy constant gamma 4
P01871	Immunoglobulin heavy constant mu
P01876	Immunoglobulin heavy constant alpha 1
P02647	Apolipoprotein A-I
P02768	Serum albumin
P02787	Serotransferrin
P02790	Hemopexin
P02808	Statherin
P02812	Basic salivary proline-rich protein 2
P02814	Submaxillary gland androgen-regulated protein 3B
P04040	Catalase
P04080	Cystatin-B
P04406	Glyceraldehyde-3-phosphate dehydrogenase
P04745	Alpha-amylase 1
P05109	Protein S100-A8
P05164	Myeloperoxidase
P05204	Non-histone chromosomal protein HMG-17
P06733	Alpha-enolase
P07476	Involucrin
P07737	Profilin-1

P09211	Glutathione S-transferase P
P09228	Cystatin-SA
P0DOX2	Immunoglobulin alpha-2 heavy chain
P0DOX7	Immunoglobulin kappa light chain
P10599	Thioredoxin
P10909	Clusterin
P12273	Prolactin-inducible protein
P13645	Keratin, type I cytoskeletal 10
P13796	Plastin-2
P14174	Macrophage migration inhibitory factor
P15515	Histatin-1
P18669	Phosphoglycerate mutase 1
P20061	Transcobalamin-1
P22528	Cornifin-B
P22531	Small proline-rich protein 2E
P22532	Small proline-rich protein 2D
P23280	Carbonic anhydrase 6
P27482	Calmodulin-like protein 3]
P28325	Cystatin-D
P29508	Serpin B3
P30086	Phosphatidylethanolamine-binding protein 1
P31025	Lipocalin-1
P31146	Coronin-1A
P31947	14-3-3 protein sigma
P31949	Protein S100-A11
P35321	Cornifin-A
P35326	Small proline-rich protein 2A
P35527	Keratin, type I cytoskeletal 9
P40925	Malate dehydrogenase, cytoplasmic
P55058	Phospholipid transfer protein
P59665	Neutrophil defensin 1
P61626	Lysozyme C
P61769	Beta-2-microglobulin
P62328	Thymosin beta-4
P62805	Histone H4
P63104	14-3-3 protein zeta/delta
P68871	Hemoglobin subunit beta
Q01469	Fatty acid-binding protein, epidermal
Q02413	Desmoglein-1
Q07654	Trefoil factor 3
Q0KKI6	Immunoblobulin light chain (Fragment)
Q0ZCH9	Immunglobulin heavy chain variable region (Fragment)

Q14508	WAP four-disulfide core domain protein 2
Q15847	Adipogenesis regulatory factor
Q2I377	Small proline rich protein
Q4VAY2	PRB3 protein
Q53H26	Transferrin variant (Fragment)
Q53H37	Calmodulin-like skin protein variant (Fragment)
Q53HE2	Triosephosphate isomerase (Fragment)
Q562R1	Beta-actin-like protein 2
Q5H9A7	Metalloproteinase inhibitor 1
Q5T3N1	Annexin (Fragment)
Q63HM4	Uncharacterized protein DKFZp686P18250
Q68DY8	Uncharacterized protein DKFZp686I11137
Q6B823	Histone H4 (Fragment)
Q6GMX6	IGH@ protein
Q6MZM9	Proline-rich protein 27
Q6N093	Uncharacterized protein DKFZp686I04196 (Fragment)
Q6P1N4	IQGAP1 protein (Fragment)
Q6P5S2	Protein LEG1 homolog
Q6PJT4	MSN protein (Fragment)
Q6ZW64	cDNA FLJ41552 fis, clone COLON2004478, highly similar to Protein Tro alpha1 H,myeloma
Q86WA0	Hypoxia-inducible HIG-1 (Fragment)
Q8N4F0	BPI fold-containing family B member 2
Q8TAX7	Mucin-7
Q8TDL5	BPI fold-containing family B member 1
Q96DR5	BPI fold-containing family A member 2
Q9GZZ8	Extracellular glycoprotein lacritin
Q9HC84	Mucin-5B
Q9P173	PRO2275
Q9UBC9	Small proline-rich protein 3
Q9UGM3	Deleted in malignant brain tumors 1 protein
Q9UJU1	Cytovillin 2 (Fragment)
Q9UL78	Myosin-reactive immunoglobulin light chain variable region (Fragment)
Q9UL90	Myosin-reactive immunoglobulin heavy chain variable region (Fragment)
Q9UNU2	Complement protein C4B frameshift mutant (Fragment)
R4GN49	Protein S100-A2
S4R460	Immunoglobulin heavy variable 3/OR16-9 (non-functional)
S6B2A1	IgG L chain
S6BAN6	IgG L chain
S6BGD6	IgG L chain

U3PXP0	Alpha globin chain (Fragment)
V9HWC6	Peptidyl-prolyl cis-trans isomerase
V9HWK1	Triosephosphate isomerase
X6RJP6	Transgelin-2 (Fragment)
